# Sepsis Neonatorum

**Published:** 1908-09-12

**Authors:** 


### Diseases of Children.

### SEPSIS NEONATORUM.

Septic infections in the new-born are commonly due to streptococci, and, less frequently, staphlyococci, aureus and albus, either alone or conjointly. The younger the infant, the more prone it is to sepsis, because of its low resisting power and undeveloped glandular system. Premature and weakly infants are peculiarly susceptible. The infecting agent generally makes its entrance through the umbilicus; less commonly through the mucous membrane of the mouth, tonsils, naso-pharynx, or conjunctiva.

Pustules on the skin are a rare cause. A child, of healthy parentage, was born at full time, without any special difficulty, and weighed over nine pounds. She was under the charge of a skilled gynæcologist and an expert monthly nurse. All the proper precautions were adopted in the management of the child after birth, and in strict attention to cleanliness of water, flannels, soaps, baths, etc. She was fed on a mixture of one-third milk and two-thirds water, and took and digested well. On examination on the ninth day of life, the child was having attacks of spasm, chiefly of the upper limbs. The arms were flexed, raised, and the hands clenched. In a severe attack the whole body twitched. The roof of the mouth showed Bednar's aphthæ. The skin was covered with a minute pustular dermatitis, none of the pustules being larger than a common pin's head.

According to the history, the rash had appeared first on the face on the fifth day of life, and the twitching had begun on the seventh day. There was no fever. The bowels acted twice or three times daily, and the stools were not quite normal in colour. In view of the possibility of the spasm being of reflex intestinal origin, the diet was changed to milk, peptonised for ten minutes, ½ oz.; water, 6 drachms; lactose, ½ drachm; for each feed every two hours. Grey powder gr. ½ was ordered at night, and pot. brom. grs. 3, four times daily. The food was readily taken in the intervals between the spasms.

On the next afternoon the temperature rose to 105° F. and it subsequently remained persistently high, ranging between 101° F. and 103° F. for a period of two weeks, occasionally a degree or so higher, and on one occasion up to 105.2° F. In the course of another week it came down to normal. The temperature chart closely resembled that of a case of typhoid fever which had passed the fourth day. The rash cleared up in about a fortnight.

During the whole of this period the spasms persisted to a varying extent, becoming less frequent and less severe as the temperature came down to lower levels. At first the spasms were most marked in the right arm, later on the left one was more affected. Sometimes they chiefly affected the facial muscles and interfered with sucking, but they never suggested the possibility of tetanus neonatorum, and there was no risus sardonicus. In bad attacks the child would become somewhat livid, and on several occasions there was grave cause for anxiety. They ceased entirely in five weeks, and have not recurred up to the present, seven weeks later.

Throughout the whole illness, with comparatively short intervals, the child took food readily as long as the spasm did not interfere with sucking. The muscles of deglutition were not involved. At times she had to be spoon-fed. The same food was given throughout, the amount and strength being increased according to the digestive capacity and appetite. During the first week of the illness there was a loss in weight of 11oz., and in the following four weeks there were successive gains of 1, 7, 6, and 4oz.

The interest in this case lies in its causation, the possible sources of infection, the diagnosis of the nature of the spasms, the prognosis, and the treatment. As stated above, there was no obvious explanation of the source of the skin infection. Possibly the skin of the face had been too vigorously washed at birth, and had become infected by wandering staphylococci, which subsequently obtained access to the general circulation and set up general sepsis. It is unlikely that the patches on the roof of the mouth, had been the source of entry, for the mouth otherwise appeared quite healthy, and the characters of the skin rash were much more suggestive of a superficial infection there.

The spasms were due to the action of the toxins on the central nervous system, more especially on the motor regions. Possibly there was a true encephalitis in this situation. Certainly there was no meningitis. That the mischief was not limited to the motor region was indicated by the dull, somewhat somnolent appearance of the child, who, for many days, took little or no interest in her surroundings. For this reason the prognosis was somewhat doubtful as to complete recovery. Fortunately at present the child seems bright, intelligent, and is growing rapidly. As regards recovery, a favourable view was taken on the grounds that the affection was staphylococcal, that the child took food well, and that she never seemed so ill as might have been expected from the severity of the spasms and the fever. In the treatment of the case, rest, quiet, darkness, limited handling, and careful feeding were insisted on. Hot baths, with cold to the head, were given for restlessness, sleeplessness, and spasm. They had a decidedly soothing effect.

After trying bromides for a few days without benefit, phenazone was given in half-grain doses every four hours. For a few days it apparently did good, the spasms being less frequent; but then they became worse, so minim doses of tinct. opii were given every six hours with distinct effect on the sleeplessness but not on the spasms. After two days of this drug, the medicine was changed to chloral grs. 2, pot. brom. grs. 3, every four hours, given in double doses per rectum, every four to six hours, according to how it was retained, and to the effect on the child. This proved the most efficacious treatment, in conjunction with hot baths, and a few minims of brandy in each feed. Chloral is, perhaps, the most reliable drug in the treatment of spasmodic affections in babies. The successful result in this case was partly due to the treatment and largely to the thorough and efficient way in which the nurses carried it out.

